# Energy Metabolism Under Stress: Late‐Stage Leigh Syndrome Reveals Profound Cardiometabolic Perturbations in *Ndufs4* KO Mice

**DOI:** 10.1002/jimd.70142

**Published:** 2026-01-14

**Authors:** Karin Terburgh, Nastassja Sweeney, Roan Louw

**Affiliations:** ^1^ Biomedical and Molecular Metabolism Research (BioMMet), Faculty of Natural and Agricultural Sciences North‐West University Potchefstroom South Africa

**Keywords:** complex I deficiency, heart metabolism, Leigh syndrome, *Ndufs4* knockout mice

## Abstract

The deficiency of mitochondrial complex I (CI), a key regulator of cellular energy homeostasis and metabolic flexibility, is a prevalent driver of cardiovascular pathology in mitochondrial disorders. The *Ndufs4* knockout (KO) mouse model of Leigh syndrome (LS), which lacks a critical CI subunit, exhibits severe cardiac abnormalities secondary to encephalomyopathy. However, the metabolic basis of LS‐associated cardiac dysfunction remains poorly understood. This study aims to evaluate how whole‐body CI deficiency affects cardiac bioenergetics and metabolism in late‐stage *Ndufs4* KO mice. We assessed respiratory chain enzyme activities and oxygen consumption rates using kinetic spectrophotometric assays and high‐resolution respirometry, respectively, in mitochondria isolated from *Ndufs4* KO and wild‐type mouse hearts. Cardiometabolic profiling was performed on a well‐powered cohort, employing untargeted GC‐TOFMS, ^1^H‐NMR and semi‐targeted LC‐MS/MS. *Ndufs4* KO hearts showed a 98.9% reduction in CI activity and a 63.9% decline in CI‐driven respiration, halving CI's contribution to combined CI + II respiration and prompting a shift toward CII‐driven respiration. Cardiometabolic profiles revealed significant reductions in energy‐generating substrates, including long‐chain fatty acids, glucose, lactic acid and 3‐hydroxybutyric acid, along with lower levels of anaplerotic amino acids and TCA cycle intermediates, particularly succinic acid. Additionally, profound disruptions were observed in dimethylglycine, glutamic acid and lysine metabolism. We conclude that whole‐body CI deficiency results in severe cardiac bioenergetic and metabolic dysregulation, characterised by reduced CI‐dependent respiration and extensive substrate reduction across multiple metabolic pathways. These findings underscore the metabolic vulnerability of the CI‐deficient heart and suggest potential therapeutic targets for managing cardiomyopathy in mitochondrial disease.

## Graphical abstract

**Cardiac bioenergetic and metabolic perturbations in late‐stage Leigh syndrome**. Whole‐body NDUFS4 loss severely impairs cardiac complex I activity and respiration, shifting reliance to complex II and reducing glycolytic, TCA, and amino acid‐derived energy‐generating substrates in the heart. These findings reveal profound cardiometabolic vulnerability underlying cardiac manifestations of Leigh syndrome.

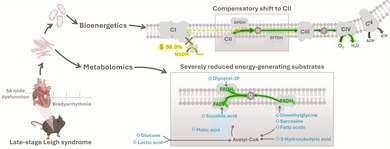

**Abbreviations**
ADP: adenosine diphosphate
AMP: adenosine monophosphate
ATP: adenosine triphosphate
BAIBA: 3‐aminoisobutyric acid
BCAA: branched‐chain amino acids
BIOPS: biopsy preservation solution
BSA: bovine serum albumin
BSTFA: N,O‐bis(trimethylsilyl)trifluoroacetamide
CI: complex I
CII: complex II
CIII: complex III
CIV: complex IV
CS: citrate synthase
DMG: dimethylglycine
EGTA: ethylene glycol tetraacetic acid
ETF: electron‐transferring flavoprotein
FADH_2_
: flavin adenine dinucleotide (reduced)
GABA: gamma‐aminobutyric acid
GC‐TOFMS: gas chromatography‐time of flight mass spectrometry
GSH: glutathione
IMP: inosine monophosphate
KO: knockout
LC‐MS/MS: liquid chromatography‐mass spectrometry/mass spectrometry
LS: Leigh syndrome
mTORC: mechanistic target of rapamycin complex
NAD^+^
: nicotinamide adenine dinucleotide (oxidised form)
NAD(P)H: nicotinamide adenine dinucleotide (phosphate)
NADPH: nicotinamide adenine dinucleotide phosphate
NAG: *N*‐acetylglutamic acid
Ndufs4: NADH dehydrogenase (ubiquinone) iron–sulfur protein 4
NMR: nuclear magnetic resonance
OXPHOS: oxidative phosphorylation
PCA: principal component analysis
QC‐CV: quality control coefficient of variation
ROS: reactive oxygen species
SANPCs: sinoatrial node pacemaker cells
TCA: tricarboxylic acid (cycle)
TMCS: trimethylchlorosilane
TSP‐d4: 3‐(trimethylsilyl)‐propionic‐2,2,3,3‐d4 acid
WT: wild‐type

## 1 Introduction

Leigh syndrome (LS), the most common paediatric manifestation of mitochondrial disease, is characterised by progressive neurodegeneration and bilateral, symmetric necrotic lesions of the brainstem and basal ganglia. Genetically, LS is highly heterogeneous, with pathogenic variants identified in over 75 nuclear and mitochondrial genes involved in oxidative phosphorylation (OXPHOS) and related pathways of energy metabolism [1, 2, 3]. The resulting bioenergetic deficiency, particularly in specific vulnerable neuronal populations, underlies the characteristic neuropathology of LS [4]. Although LS primarily presents with central nervous system involvement, clinical features vary, with the pathology often extending to peripheral tissues. Cardiac involvement is increasingly recognised as a significant feature of the disease, strongly associated with a poor prognosis. Reports indicate variable cardiac manifestation, with 18%–21% of LS patients developing severe complications, including hypertrophic cardiomyopathy, conduction defects and pericardial effusion [1, 5].

Mitochondrial OXPHOS defects, particularly complex I (CI) dysfunction, are central to the pathology of both LS‐associated cardio‐encephalomyopathy [6, 7] and a variety of common cardiac conditions, including age‐related cardiac decline [8], diabetes‐related cardiovascular disease, hypertrophy, arrhythmias and heart failure [9, 10, 11]. CI (NADH:ubiquinone oxidoreductase) is vital for mitochondrial function, driving ATP production as the primary electron donor to the ubiquinone pool and key proton‐pumping complex in the respiratory chain. Beyond its role in energy generation, this multimeric enzyme complex is critical for cellular redox homeostasis, recycling mitochondrial NAD^+^ and serving as a major source of reactive oxygen species (ROS). Accordingly, deficiency of CI can profoundly disrupt cellular bioenergetics and metabolic homeostasis [12, 13].

The assembly and stability of CI, both as an individual complex and within respiratory supercomplexes, are critically dependent on the accessory subunit, NDUFS4 [14, 15, 16, 17]. Loss of NDUFS4 [NADH dehydrogenase (ubiquinone) iron–sulfur protein 4]—a mutational hotspot for CI deficiency [1]—also results in near‐complete depletion of NDUFA12, which interacts with NDUFS4 to bridge the N‐ and Q‐modules of CI [15]. This disruption weakens the association of the N‐module with the remainder of the complex, resulting in mutant CI assemblies that either retain the assembly factor NDUFAF2 or incorporate the NDUFS6 subunit [17]. These compositional alterations, including both subunit loss and gain, impair CI maturation and stability, culminating in severe CI deficiency, mitochondrial dysfunction and the clinical manifestation of LS [17, 18, 19]. *NDUFS4*‐related LS, while characterised by neurological dysfunction, demonstrates significant cardiac involvement with hypertrophic cardiomyopathy documented (Table S1) in approximately 25% (8 of 32) of described cases [1, 20, 21, 22, 23, 24, 25, 26, 27, 28, 29, 30, 31, 32, 33, 34, 35, 36, 37].

The *Ndufs4* knockout (KO) mouse model of LS has been pivotal in studying the tissue‐specific consequences of CI deficiency [19]. These mice recapitulate key aspects of human LS, including progressive neurodegeneration and symmetrical brain lesions, with systemic impairments becoming pronounced as the disease progresses. Late‐stage symptoms include severe weight loss, ataxia, hypothermia, bradycardia, reduced respiratory rates and low arterial oxygenation, typically leading to early mortality between 55 and 75 days postnatal [38, 39, 40].

Cardiac involvement in this model has been increasingly documented, though findings vary across studies [41]. Myocyte‐specific (Ckmm‐NLS) *Ndufs4* KO mice display heart enlargement without clinical symptoms for up to 12 months [42], while another study reports hypertrophic cardiomyopathy without signs of oxidative stress [43]. Cardiomyocyte‐specific (αMHC) *Ndufs4* KO mice appear healthy at rest but progress to heart failure under physiological stressors, with this decline linked to NAD^+^ deficiency‐driven protein hyperacetylation [44]. In contrast, whole‐body *Ndufs4* KO mice do not develop cardiomyopathy but exhibit severe bradyarrhythmia with sinoatrial nodal dysfunction [6, 7], a defect specifically attributed to *Ndufs4* deficiency in conduction cells (HCN4) [6]. These abnormalities were mechanistically tied to NAD^+^ deficiency‐driven protein hyperacetylation and oxidative stress, with interventions like an NAD^+^ precursor, mitochondrial antioxidant and protective peptide alleviating bradyarrhythmia and extending lifespan [6, 7].

Given the heart's critical reliance on efficient mitochondrial OXPHOS, understanding how CI deficiency disrupts cardiac metabolism is essential for identifying potential therapeutic targets. We hypothesised that CI dysfunction in whole‐body *Ndufs4* KO mice impairs mitochondrial respiration in cardiac tissue, triggering a compensatory increased reliance on CII activity and leading to widespread substrate depletion, which ultimately drives cardiac bioenergetic dysfunction in LS.

To test this hypothesis, we performed an integrated assessment of cardiac mitochondrial function and metabolism in late‐stage *Ndufs4* KO and wild‐type (WT) mice. We combined enzymatic assays, high‐resolution respirometry and multiplatform metabolomics to characterise the extent of CI impairment and its metabolic consequences. Metabolic analyses were conducted in a well‐powered cohort (*n* ≥ 14 per group) with all animals synchronised by disease stage, circadian phase and fasting state. Building on our previous work delineating tissue‐specific metabolic disruptions in this same cohort [45, 46, 47, 48], we identify both pronounced and subtle cardiometabolic shifts driven by whole‐body CI deficiency that can be directly compared to other tissues and offer new insights into the disease's systemic impact.

## 2 Methods

### 2.1 Animal Handling and Tissue Collection

*Ndufs4* KO and WT mice were generated from heterozygous breeding pairs (B6.129S4‐*Ndufs4* ^tm1.1Rpa^/J, The Jackson Laboratory, Bar Harbor, ME, USA; JAX #027058) and maintained under standard conditions (12:12 h light/dark cycle, 22°C ± 1°C, 55% ± 10% humidity) with ad libitum access to Rodent Breeder chow (#RM1845, Lab Chef, Nutritionhub, SA) and water at the North‐West University Preclinical Drug Development Platform (SAVC reg. #FR15/13458). All experimental procedures were approved by the NWU Animal Care, Health and Safety Research Ethics Committee (#NWU‐00430‐21‐A5, #NWU‐00001‐15‐A5 and #NWU‐0509‐20‐A1). Only male mice were used in this study to minimise sex‐related metabolic variability due to known differences in metabolic profiles, hormonal cycles and tissue‐specific metabolism between sexes [49, 50]. Mice were euthanised at the late stage of LS (45–50 days postnatal) via cervical dislocation, performed at a consistent time each day. Heart tissues were rapidly excised and either processed immediately for mitochondrial isolation (*n* ≥ 3 biological replicates per group) or snap‐frozen in liquid nitrogen and stored at −80°C for metabolic analysis (*n* ≥ 14 biological replicates per group). For metabolic profiling, heart tissues were collected following a 12‐h fasting period.

### 2.2 Isolation of Cardiac Mitochondria

Mitochondria were isolated from heart tissue using differential centrifugation as previously described [51], with all steps performed on ice. After removing adipose tissue, major vessels and atria, the heart was placed in biopsy preservation solution (BIOPS), cleaned of blood clots and minced (40–75 mg) in 1 mL BIOPS. The tissue was then homogenised in 2 mL isolation buffer (75 mM sucrose, 225 mM mannitol, 1 mM EGTA, 2.5 mg/mL BSA and 0.5 mg/mL subtilisin, pH 7.4) with ~20 strokes using a 7 mL Dounce tissue grinder with small clearance pestle (KIMBLE, Vineland, NJ, USA). Thereafter, 3 mL isolation buffer was added and the homogenate was centrifuged at 800*g* (10 min, 4°C) to remove debris, followed by 10 000*g* (10 min, 4°C) to pellet mitochondria. The pellet was then resuspended in 2.5 mL isolation buffer without subtilisin, centrifuged again (10 000*g*, 10 min, 4°C) and the final mitochondrial pellet resuspended in 200 μL suspension buffer (75 mM sucrose, 225 mM mannitol, 1 mM EGTA).

### 2.3 Measurement of Respiratory Chain Enzyme Activity

Enzyme assays for respiratory chain complexes I‐IV and citrate synthase (CS) were performed spectrophotometrically at 37°C using a Synergy HT microplate reader (BioTek Instruments, Winooski, VT, USA) as previously described [52, 53, 54, 55]. Mitochondria were subjected to three freeze–thaw cycles before enzyme activity was measured in triplicate for each biological repeat. Data analysis was done using Gen5 Data Analysis software version 1.11.5, with enzyme activities normalised to units of CS (UCS in μmol/min/mg protein) as a marker of mitochondrial content.

### 2.4 Respirometry

Mitochondrial respiration was measured at 37°C using an O2k respirometer (Oroboros Instruments, Innsbruck, Austria) following the SUIT‐008 O2 mt D026 protocol. Mitochondria were suspended in MiR05 buffer, and respiration was assessed through stepwise additions of substrates and inhibitors for isolated and combined CI‐ (pyruvic acid, malic acid, ADP and glutamic acid) and CII‐driven (succinic acid and rotenone) respiration. Respiration rates were corrected for instrumental background and residual oxygen consumption, and normalised to units of CS (UCS in μmol/min/mg protein) as a marker of mitochondrial content. Control ratios for CI and CII were calculated by dividing the respiration rate of each complex in isolation by the total respiration rate (CI + II) when active simultaneously. Control ratios were expressed as CI/(CI + II) and CII/(CI + II), respectively, to evaluate the relative contributions of each complex.

### 2.5 Metabolic Profiling

Metabolic profiling was performed according to protocols previously established at our facilities [47], with minor modifications detailed in the Supporting Information. Briefly, pre‐weighed heart tissue (40–75 mg) was subjected to single‐phase Bligh‐Dyer extraction using a 3:1:1 ratio of methanol/water/chloroform (HPLC grade) [56]. After extraction, the tissue was homogenised using three 3 mm tungsten carbide beads per sample (Qiagen, Hilden, Germany) and a Retsch MM 400 vibration mill (Retsch GmbH, Haan, Germany) at 30 Hz for 2 min. During extraction, the water added contained internal standards (1 μg/mg tissue), including *N*,*N*‐dimethyl‐l‐phenylalanine, 3‐phenybutyric acid and norleucine.

Metabolic extracts were analysed using three complementary techniques, that is, via untargeted ^1^H‐NMR spectroscopy on a 500 MHz Bruker Avance III HD spectrometer (Bruker BioSpin, Billerica, MA, USA), untargeted GC‐TOFMS on an Agilent 7890A GC system (Agilent Technologies, Santa Clara, CA, USA) coupled to a Pegasus HT TOF mass analyser (LECO Corporation, St. Joseph, MI, USA), and semi‐targeted LC‐MS/MS analysis of amino acids, amino acid derivatives and acylcarnitines (Table S2) on an Agilent 6470 system. Prior to analysis, NMR extracts were resuspended in HPLC‐grade water and mixed with TSP‐d4 buffer. For LC‐MS/MS, the extracts were butylated with 1‐butanol:acetyl chloride (4:1, v/v), dried and reconstituted in a 50:50 (v/v) mixture of water and acetonitrile, containing 0.1% formic acid. For GC‐TOFMS, the extracts were oximated with methoxyamine hydrochloride in pyridine (20 mg/mL) and silylated with BSTFA containing 1% TMCS.

Data were baseline‐corrected and processed in software specific to each platform, with manual review for accurate peak identification. For GC‐TOFMS analysis, data acquisition and peak extraction were performed using LECO ChromaTOF software version 4.5x, with peak alignment conducted via the Statistical Compare module. All detected features were statistically evaluated. Among features showing significant differences between groups, only those that could be confidently annotated were reported and interpreted. Peaks were annotated by matching spectral and retention time data to commercial and in‐house libraries, using a similarity threshold of ≥ 80%. LC‐MS/MS data were acquired and processed using Agilent MassHunter Workstation software (version B02.01 for acquisition; version B06.00 for processing). Metabolite identification was achieved by monitoring two unique transitions per metabolite, allowing for both spectral and retention time confirmation. Validation was conducted by analysing a pure reference standard mixture within the same batch. For ^1^H NMR, spectra were processed using Bruker Topspin software version 3.5, with further spectral analysis and metabolite identification performed in Bruker AMIX software version 3.9.14. NMR spectra were initially binned, and all bins were statistically analysed to identify spectral regions exhibiting statistically significant differences between groups. Significant bins were inspected to identify individual peaks, which were then assigned metabolite annotations (using 1D and 2D spectral library databases of pure compounds), reprocessed for accurate integration and metabolite concentration determination and subjected to further statistical analysis. Only confidently annotated metabolites are interpreted. Metabolites were identified from 1D spectra through comparison with commercial and in‐house libraries, and their identities were confirmed using 2D ^1^H‐^1^H correlation and J‐resolved spectroscopy for maximum confidence.

The data were then preprocessed in Microsoft Excel (Microsoft Corporation, Redmond, WA, USA) to remove unreliable features with high missing values (modified 80% rule); high QC‐CV values (> 25% for semi‐targeted data and > 50% for untargeted data) and high variance (Fisher ratio < 0.05 [57]). Features were normalised to internal standards, which reflects tissue mass, with the data subsequently log transformed and auto scaled.

### 2.6 Data Analysis

Two‐tailed Student's *t*‐tests were used for statistical comparisons, with Bonferroni–Holm corrections applied for multiple testing where necessary. Effect sizes (Cohen's D values) were calculated to assess practical significance. Differences with *p* < 0.05 and *d* > 0.5 were considered significant. Bioenergetic data were visualised with BoxPlotR (Spitzer Lab, University of Vienna, Austria [58]), and metabolic data were analysed using MetaboAnalyst software version 5.0 (Xia Lab, McGill University, Montreal, QC, Canada [59, 60]) for principal component analysis (PCA) and hierarchical clustering. Multivariate analyses of metabolic data were conducted on combined datasets, with outlier samples and duplicate metabolites removed.

## 3 Results

### 3.1 Respiratory Chain Enzyme Activity and Respirometry

**FIGURE 1:**
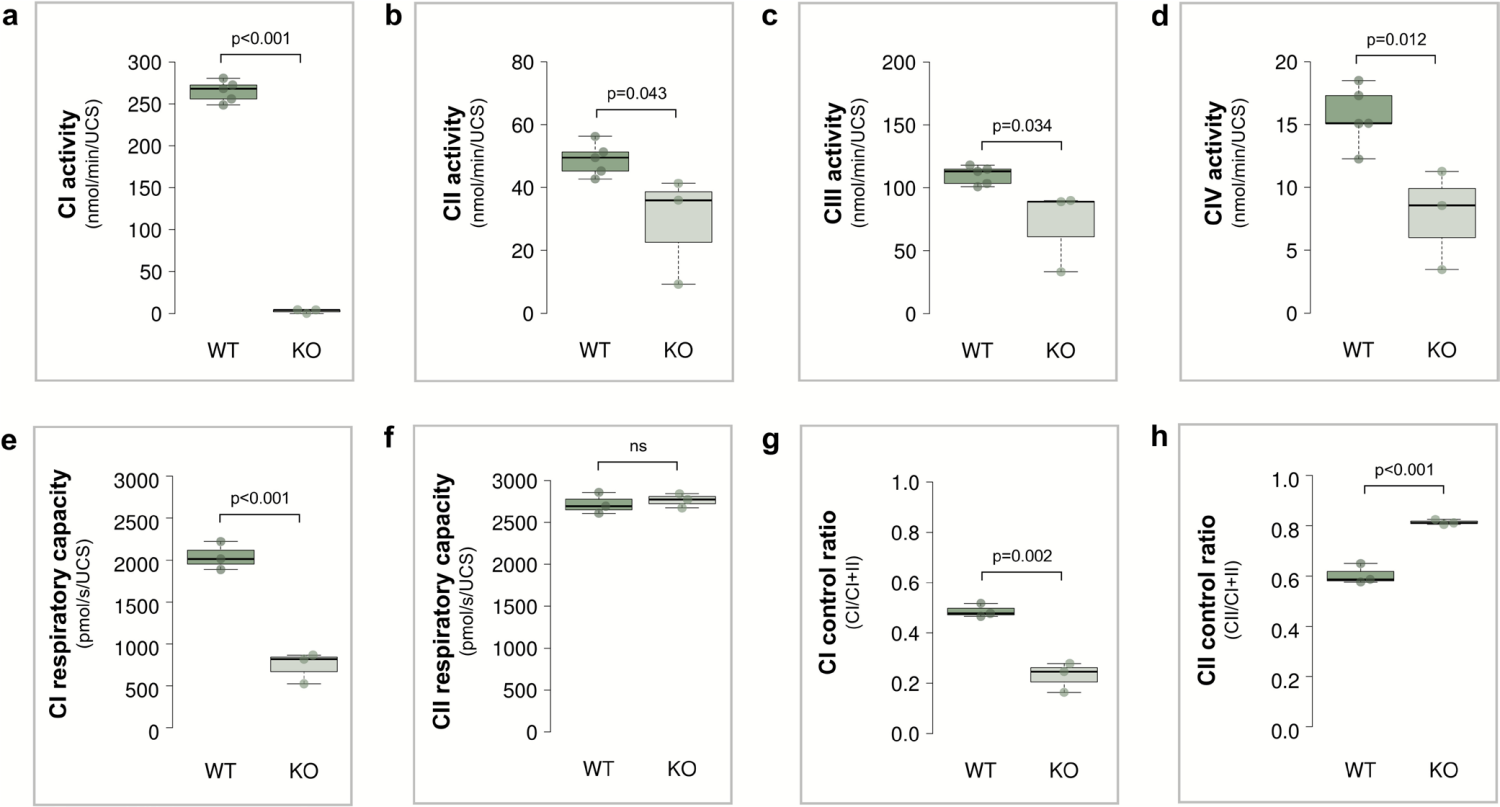
Mitochondrial respiratory chain enzyme activity and respiration in the heart of *Ndufs4* KO versus WT mice. (a–d) Maximal enzyme activities of complexes I–IV in mouse heart mitochondria, normalised to units of citrate synthase (UCS). Each data point represents the average of triplicate measurements from WT (*n* = 5) and KO (*n* = 3) mice. Respiratory capacity for CI (e) and CII (f) in heart mitochondria from WT (*n* = 3) and KO (*n* = 3) mice, normalised to UCS. Control ratios for CI (g) and CII (h). CI control ratios were calculated by dividing oxygen consumption from CI‐driven respiration (with pyruvic acid, malic acid, ADP and glutamic acid) by that from CI + II respiration (with succinic acid). Similarly, CII control ratios were determined by dividing CII‐driven respiration (with succinic acid, uncoupler and rotenone) by CI + CII respiration (with succinic acid and uncoupler). Statistical analysis was performed using a two‐way Student's *t*‐test, with box plots showing minimum, maximum, interquartile range and median values. CI, complex I; CII, complex II; CIII, complex III; complex IV; KO, knockout; UCS, units of citrate synthase; WT, wild‐type.

Respiratory chain enzyme activities and mitochondrial respiration were analysed in heart mitochondria from *Ndufs4* KO (*n* = 3) and WT (*n* ≥ 3) mice (Figure 1). Enzyme activities, measured in mitochondrial preparations after membrane disruption, showed a severe 98.9% reduction in rotenone‐sensitive CI activity (Figure 1a *p* < 0.001, *d* = 20.51), along with decreased activities of CII (41.1%, Figure 1b, *p* = 0.043, *d* = 1.17), CIII (35.8%, Figure 1c, *p* = 0.034, *d* = 1.21) and CIV (50.4%, Figure 1d, *p* = 0.012, *d* = 1.99).

In contrast, high‐resolution respirometry performed on intact, isolated mitochondria revealed a 63.9% reduction in isolated CI‐driven respiration (Figure 1e; *p* < 0.001, *d* = 7.07), whereas isolated CII‐driven respiration remained unaltered (Figure 1f). Analysis of control ratios indicated a marked reduction in CI's contribution to combined respiration (CI control ratio decreasing from 0.49 in WTs to 0.23 in KOs; Figure 1g; *p* = 0.002, *d* = 4.36), accompanied by a compensatory increase in CII electron input (CII control ratio increasing from 0.60 in WTs to 0.81 in KOs; Figure 1h; *p* < 0.001, *d* = 5.28) to maintain a balanced convergent electron flow between the two complexes.

### 3.2 Metabolic Profiling

**FIGURE 2:**
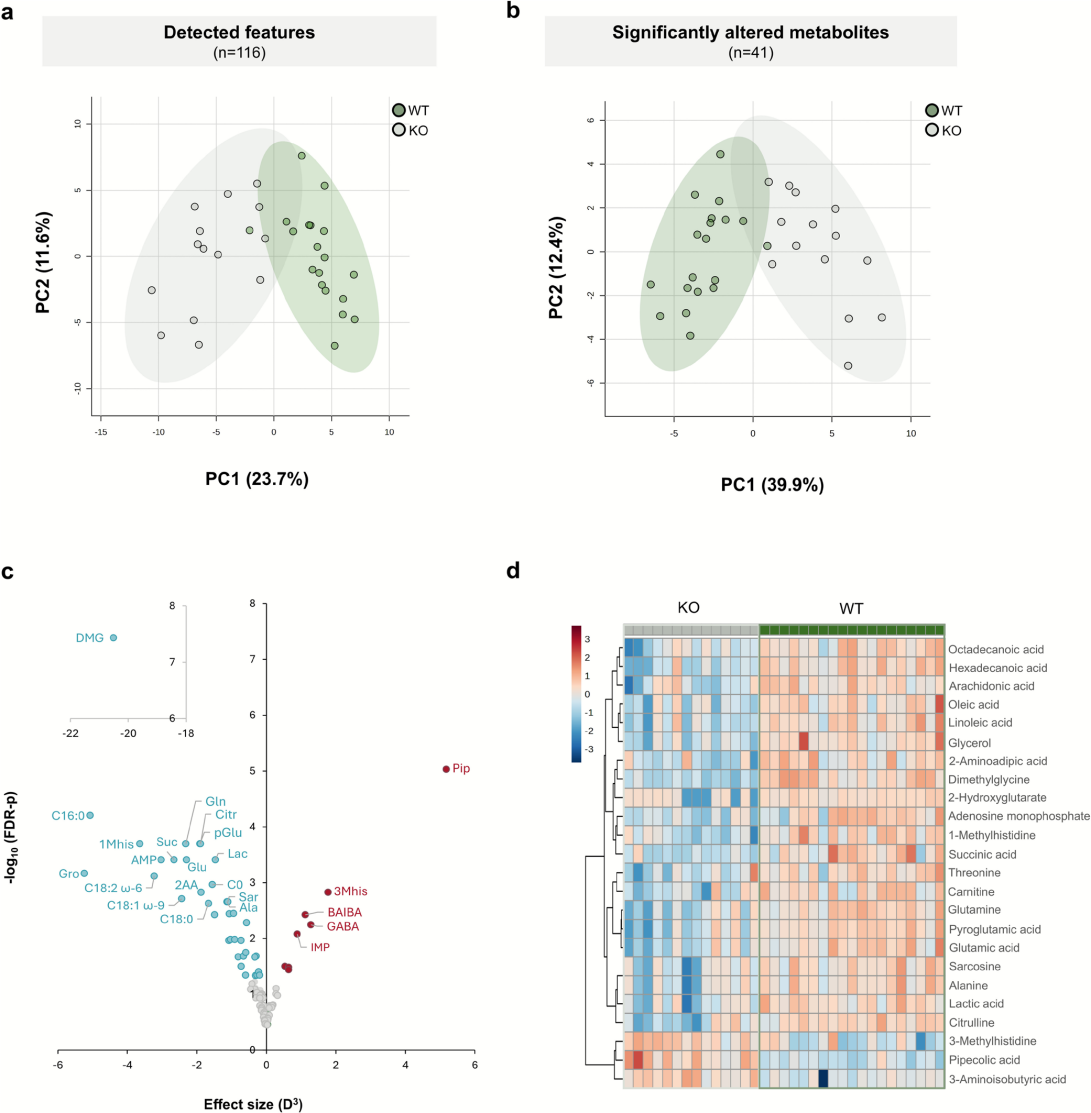
Metabolic profiling of the heart of *Ndufs4* KO versus WT mice. (a, b) Principal component analysis (PCA) of metabolites detected in WT and KO mouse hearts using LC‐MS/MS, GC‐TOFMS and ^1^H‐NMR spectroscopy shown before (a) and after (b) feature selection. The first two principal components are plotted, with ellipses representing 95% confidence intervals for each group. Data were log‐transformed and auto scaled (*n* = 14 KO, *n* = 19 WT). (c) Volcano plots displaying the relationship between statistical significance (−log10 FDR‐corrected *p* values) and effect size (cubed Cohen's *D*) for metabolites differentiating *Ndufs4* KO from WT hearts. Metabolites significantly reduced or elevated in KO compared to WT are represented by blue and red filled circles, respectively (FDR‐corrected *p* < 0.05 and *D* ≥ 0.5). Non‐significant features are shown in grey. For untargeted GC data, unknown features were excluded. (d) Hierarchical clustering heatmap illustrating the relative abundance of metabolites across samples. Clustering was conducted using the Euclidean distance metric and Ward's linkage method, focusing on the 24 metabolites with the lowest *t*‐test *p* values. For untargeted GC data, unknown features were excluded. 1Mhis, 1‐methylhistidine; 2AA, 2‐aminoadipic acid; 3Mhis, 3‐methylhistidine; Ala, alanine; AMP, adenosine monophosphate; BAIBA, 3‐aminoisobutyric acid; C0, carnitine; C16:0, palmitic acid; C18:0, stearic acid; C18:1 *ω*‐9, oleic acid; C18:2 *ω*‐6, linoleic acid; Citr, citrulline; DMG, dimethylglycine; GABA, 4‐aminobutyric acid; Glu, glutamic acid; Gln, glutamine; Gro, glycerol; Lac, lactic acid; pGlu, pyroglutamic acid; Pip, pipecolic acid; Sarc, sarcosine; Suc, succinic acid.

After filtering the data and removing duplicate compounds from the combined datasets, 116 features remained. Among these, 41 metabolites were identified as being significantly altered between the *Ndufs4* KO and WT heart samples (Table 1). Multivariate PCA (Figure 2a,b) revealed distinct cardiac metabolic profiles between *Ndufs4* KO and WT mouse heart samples. Genotypic differences accounted for 35.3% of the variance in the first two principal components (PC1 = 23.7%, PC2 = 11.6%) when all detected features were analysed and for 50.4% of the variance (PC1 = 39.7%, PC2 = 10.7%) when only significantly altered metabolites were included. A volcano plot (Figure 2c) was constructed to visualise these metabolites (FDR‐*p* < 0.05, *D* > 0.5). The *x*‐axis represents the effect size (D^3^) between genotypes, while the y‐axis shows the −log_10_ of the FDR‐corrected *p* values. A heatmap was generated using hierarchical clustering to visualise the differential abundance of the 24 metabolites with the lowest *p* values between genotypes (Figure 2d). Rows represent metabolites, and columns represent individual samples. As evident from these graphs, most metabolites were significantly decreased in *Ndufs4* KO hearts compared to WTs.

**TABLE 1:** Significantly altered metabolites discriminating between the *Ndufs4* KO and WT mouse heart.

Metabolite	Abbreviation	Platform	ID level^a^	*t*‐test *p*	FDR‐*p*	Effect size	Direction in KO^b^	Platform confirmed^c^
Adenosine monophosphate	AMP	NMR	1	1.19E‐04	3.90E−04	1.45	↓	
Succinic acid	Suc	NMR	1	5.81E−05	3.90E−04	1.39	↓	GC
Glutamic acid	Glu	NMR	1	1.09E−04	3.90E−04	1.32	↓	GC
Carnitine	C0	NMR	1	5.89E−04	1.08E−03	1.16	↓	
Lactic acid	Lac	NMR	1	1.42E−04	3.90E−04	1.14	↓	GC
α‐Glucose	Gluc	NMR	1	1.51E−02	2.08E−02	0.66	↓	
Inosine monophosphate	IMP	NMR	1	5.32E−03	8.35E−03	0.96	↑	
Dimethylglycine	DMG	LC	1	1.02E−09	3.67E−08	2.74	↓	
1‐Methylhistidine	1Mhis	LC	1	2.97E−05	2.02E−04	1.54	↓	
Glutamine	Gln	LC	1	3.36E−05	2.02E−04	1.32	↓	
Pyroglutamic acid	pGlu	LC	1	2.94E−05	2.02E−04	1.24	↓	GC
Citrulline	Citr	LC	1	2.93E−05	2.02E−04	1.24	↓	
2‐Aminoadipic acid	2AA	LC	1	2.95E−04	1.49E−03	1.23	↓	
Hexanoylcarnitine	C6	LC	1	1.36E−03	3.75E−03	1.14	↓	
Alanine	Ala	LC	1	5.74E−04	2.20E−03	1.04	↓	NMR, GC
Sarcosine	Sarc	LC	1	6.12E−04	2.20E−03	1.03	↓	
Butyrylcarnitine	C4	LC	1	5.44E−03	1.09E−02	1.02	↓	
Threonine	Thr	LC	1	1.11E−03	3.63E−03	1.02	↓	GC
Hexadecanoylcarnitine	C16	LC	1	4.35E−03	1.04E−02	1.01	↓	
Propionylcarnitine	C3	LC	1	4.66E−03	1.05E−02	0.97	↓	
Octadecanoylcarnitine	C18	LC	1	1.25E−02	2.15E−02	0.93	↓	
4‐Hydroxyproline	4Hpro	LC	1	5.17E−03	1.09E−02	0.90	↓	
Tetradecanoylcarnitine	C14	LC	1	1.16E−02	2.08E−02	0.87	↓	
*N*‐Acetylglutamic acid	NAG	LC	1	9.46E−03	1.79E−02	0.84	↓	
3‐Aminoisobutyric acid	BAIBA	LC	1	1.36E−03	3.75E−03	1.04	↑	
4‐Aminobutyric acid	GABA	LC	1	2.22E−03	5.71E−03	1.08	↑	
3‐Methylhistidine	3Mhis	LC	1	3.31E−04	1.49E−03	1.21	↑	
Pipecolic acid	Pip	LC	1	5.18E−07	9.33E−06	1.73	↑	GC
Glycerol	Gro	GC	2	2.42E−05	6.86E−04	1.74	↓	
Palmitic acid	C16:0	GC	2	1.47E−06	6.27E−05	1.72	↓	
Linoleic acid	C18:2 *ω*‐6	GC	2	3.60E−05	7.65E−04	1.48	↓	
Oleic acid	C18:1 *ω*‐9	GC	2	2.05E−04	1.94E−03	1.35	↓	
Stearic acid	C18:0	GC	2	2.77E−04	2.35E−03	1.18	↓	
Arachidonic acid	C20:4 *ω*‐6	GC	3	6.31E−04	3.57E−03	0.98	↓	
Malic acid	Mal	GC	2	8.90E−03	3.15E−02	0.90	↓	
Glycerol‐1‐monopalmitate	MG	GC	3	1.62E−02	4.56E−02	0.84	↓	
2‐Hydroxyglutaric acid	2HG	GC	2	1.17E−03	5.24E−03	0.83	↓	
Putrescine	Put	GC	3	5.33E−03	2.16E−02	0.69	↓	
Uridine	Urd	GC	2	1.80E−02	4.63E−02	0.68	↓	
3‐Hydroxybutyric acid	3HB	GC	2	1.68E−02	4.56E−02	0.64	↓	
α‐Linolenic acid	C18:3 *ω*‐3	GC	3	1.30E−02	3.95E−02	0.61	↓	

^a^ Confidence levels of metabolite identification are based on the classification system proposed by Schymanski and colleagues [61].

^b^ Arrows indicate the direction of change in metabolite levels in KO relative to WT (↑ = increase, ↓ = decrease).

^c^ Additional analytical platform where the metabolite was also detected and found to be significantly altered.

### 3.3 Pathway Mapping

**FIGURE 3:**
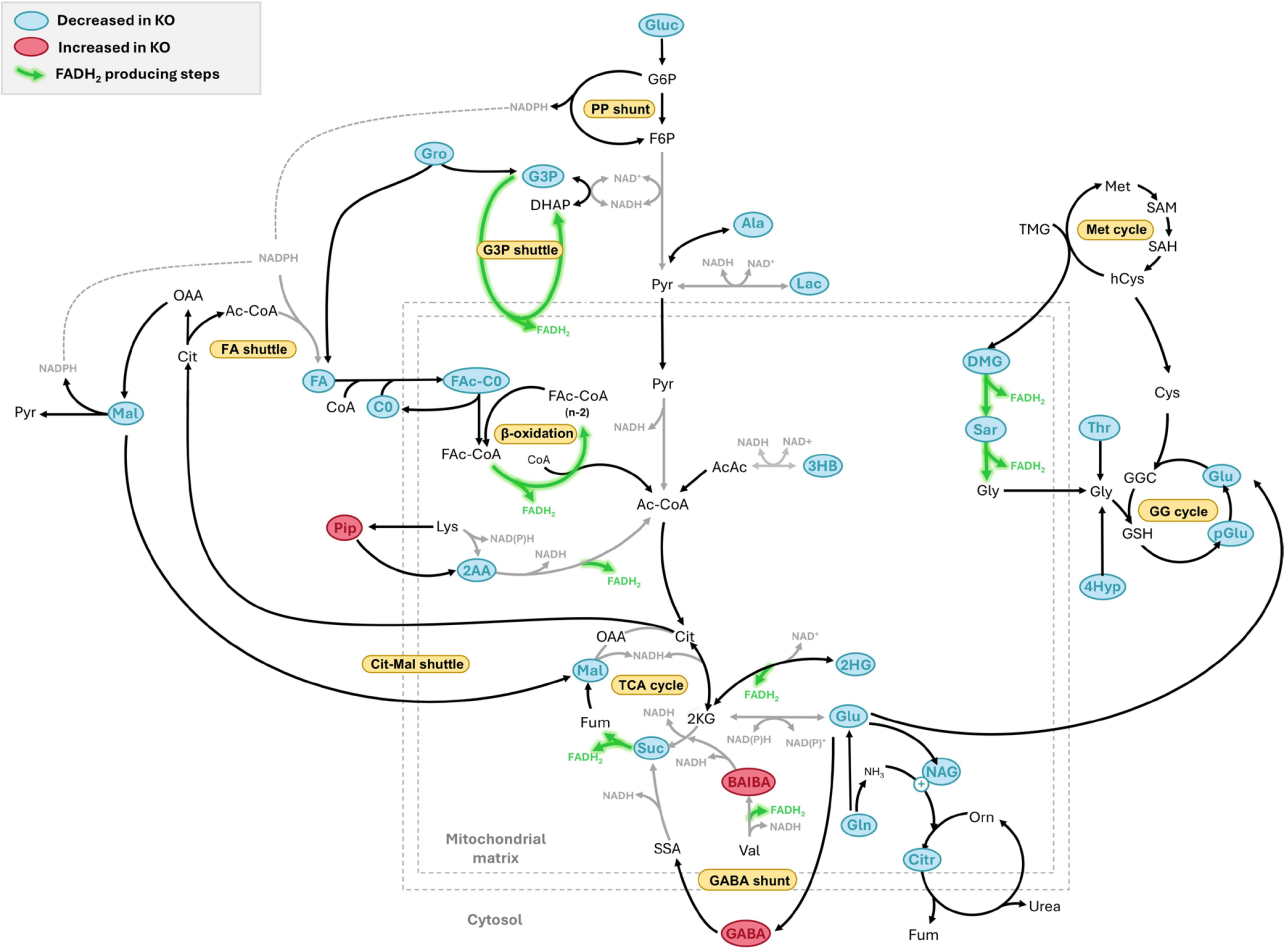
Metabolic pathway perturbations in *Ndufs4* KO vs WT hearts. Significant alterations were observed in both primary and alternative pathways of energy metabolism, including those involving fatty acids, lactic acid, glucose, ketone bodies and anaplerotic amino acids. Many of the affected pathways are sensitive to redox state alterations in NAD(P)H/NAD(P)^+^ ratios and/or involve steps that produce FADH_2_. 2AA, 2‐aminoadipic acid; 2HG, 2‐hydroxyglutaric acid; 3HB, 3‐hydroxybutyric acid; AcAc, acetoacetic acid; Ac‐CoA, acetyl coenzyme A; Ala, alanine; BAIBA, 3‐aminoisobutyric acid; C0, free carnitine; CoA, coenzyme A; Cys, cysteine; DHAP, dihydroxyacetone phosphate; DMG, dimethylglycine; F6P, fructose‐6‐phosphate; FA, fatty acid; FAc‐C0, fatty acylcarnitine; FAc‐CoA, fatty acyl coenzyme A; FADH_2_, reduced flavin adenine dinucleotide; Fum, fumaric acid; G3P, glycerol‐3‐phosphate; G6P, glucose‐6‐phosphate; GG, gamma‐glutamyl; GGC, gamma‐glutamylcysteine; Glu, glutamic acid; Gluc, glucose; Gly, glycine; Gro, glycerol; GSH, reduced glutathione; hCys, homocysteine; Lac, lactic acid; Lys, lysine; Mal, malic acid; Met, methionine; NAD(P)^+^, oxidised nicotinamide adenine dinucleotide (phosphate); NAD(P)H, reduced nicotinamide adenine dinucleotide (phosphate); NAG, *N*‐acetylglutamic acid; OAA, oxaloacetic acid; Orn, ornithine; pGlu, pyroglutamic acid; Pip, pipecolic acid; PP, pentose phosphate; Pyr, pyruvic acid; SAH, *S*‐adenosylhomocysteine; SAM, *S*‐adenosylmethionine; Sar, sarcosine; SSA, succinic semialdehyde; Suc, succinic acid; Thr, threonine; TMG, trimethylglycine (betaine); Val, valine.

The cardiometabolic profile of late‐stage diseased *Ndufs4* KO mice (Figure 3) revealed significantly decreased levels of fatty acids, including saturated (palmitic acid [C16:0] and stearic acid [C18:0]), monounsaturated (oleic acid [C18:1]) and polyunsaturated fatty acids (linoleic acid [C18:2 *ω*‐6], α‐linoleic acid [C18:3 *ω*‐3] and arachidonic acid [C20:4 *ω*‐6]). Among these, palmitic acid exhibited the most pronounced decrease, accompanied by markedly reduced levels of glycerol, the monoacylglycerol glycerol‐1‐monopalmitate, and notable reductions in glycerol‐3‐phosphate (*p* = 0.034; FDR‐*p* = 0.082, *D* = 0.62). Correspondingly, reduced levels of free carnitine (C0) and various acylcarnitines (C3, C4, C6, C14, C16 and C18) were evident in *Ndufs4* KO hearts. Together, these alterations point to significant disruptions in cardiac fatty acid transport, mitochondrial oxidation and triglyceride metabolism.

Additionally, *Ndufs4* KO hearts displayed marked decreases in other key energy‐generating substrates, including lactic acid, glucose and 3‐hydroxybutyric acid, with the lactic acid/pyruvic acid ratio significantly decreased (*p* = 0.001, *D* = 1.21). In accordance, tricarboxylic acid (TCA) cycle metabolites such as 2‐hydroxyglutaric acid (a derivative of 2‐ketoglutaric acid), succinic acid and malic acid were significantly reduced while notable decreases in fumaric acid (*p* = 0.060; FDR‐*p* = 0.116, *D* = 0.67) were also evident. These metabolic shifts extended to nucleotide metabolism, as evidenced by reduced levels of uridine and adenosine monophosphate (AMP), with a concomitant increase in inosine monophosphate (IMP).

Furthermore, some of the most pronounced alterations in *Ndufs4* KO hearts were observed in amino acid metabolism. Dimethylglycine (DMG), a choline‐derived precursor to glycine, was markedly reduced, along with its downstream metabolite, sarcosine and other glycine precursors, threonine and 4‐hydroxyproline. Conversely, the lysine catabolite, pipecolic acid, was significantly elevated in *Ndufs4* KO hearts, while its downstream metabolite, 2‐amino adipic acid, was notably decreased. 3‐Aminoisobutyric acid (BAIBA) and 4‐aminobutyric acid (GABA) were markedly elevated in *Ndufs4* KO hearts, with other metabolites related to glutamic acid and nitrogen handling also significantly diminished, including alanine, glutamine, glutamic acid, pyroglutamic acid, *N*‐acetylglutamic acid and citrulline, along with notable decreases in proline (*p* = 0.049; FDR‐*p* = 0.077, *D* = 0.69). Moreover, the levels of methylated histidine derivatives were markedly altered in *Ndufs4* KO cardiac tissue, with 1‐methylhistidine decreased and 3‐methylhistidine increased.

## 4 Discussion

Enzyme assays revealed a near‐complete loss (98.9%) of CI activity in the hearts of severely diseased whole‐body *Ndufs4* KO mice (~7 weeks old). This apparent depletion likely reflects the intrinsic instability of mutant CI rather than a true absence of catalytic activity in situ. Recent cryo‐EM analysis of NDUFS4‐deficient CI from mouse heart [17] provides structural evidence for this instability, revealing aberrant subassemblies that either retain the assembly factor NDUFAF2 or incorporate NDUFS6, alongside loss of both NDUFS4 and NDUFA12. In intact *Ndufs4* KO cardiac mitochondria, the native inner membrane and association with respiratory supercomplexes partially stabilises CI, preserving substantial residual activity [14, 17, 42]. However, disruption of the mitochondrial membrane—by freeze–thaw cycles or detergent solubilisation—destabilises mutant CI, promoting dissociation and an artefactual loss of activity [42]. This assay‐dependent variability is evident across studies with detergent permeabilised mitochondrial preparations yielding 75%–95% cardiac CI loss in (cardio)myocyte‐specific KOs [42, 44], whereas gentler conditions showed 50%–56% loss in myocyte‐specific [43] and whole‐body [14] KO models.

In addition to severe CI deficiency, *Ndufs4* KO hearts showed significantly reduced activities of CII (41.1%), CIII (35.8%) and CIV (50.4%). However, the magnitude of these reductions requires confirmation in larger cohorts given the small sample size (*n* = 3) and presence of one near‐outlier KO sample skewing the data downward. Nevertheless, these multi‐complex deficits align with findings in cardiomyocyte‐specific *Ndufs4* KO hearts (~14 weeks old), where decreased CII activity and modest reductions in CII/III and CIV coincided with impaired formation of CI‐containing supercomplexes and diminished CI activity within residual supercomplexes [42]. Together, these data point to supercomplex instability secondary to cardiac NDUFS4 loss. Beyond the heart, we have reported other (moderate) tissue‐specific alterations in CII–CIV activities, with CIII decreased by 18% in soleus but not quadriceps muscle [47], CIV increased by 10%–12% across brain regions [45] and activities in the liver unchanged [48]. These heterogeneous responses contrast with earlier reports of stable CII–IV activities in *Ndufs4* KO tissues, including the heart [14, 43, 44] and implicate supercomplex organisation and stability as potential contributors to tissue‐specific vulnerability.

Functionally, the marked reduction in cardiac CI stability resulted in a 63.9% decrease in CI‐driven respiration in *Ndufs4* KO heart mitochondria, accompanied by a 52.9% decline in CI's contribution to combined (CI + II) respiration. This indicates that the mutant enzyme retains substantial residual activity within the intact mitochondrial architecture, consistent with previous reports [42] highlighting the critical stabilising role of the inner membrane and respiratory supercomplexes in the heart. Furthermore, this CI impairment appears to trigger a compensatory shift towards CII‐driven respiration, with CII's contribution to combined respiration increasing by 34.7%, likely to support ATP production. In line with this, Adjobo‐Hermans and colleagues [15] reported increased levels of CII subunits and the CV assembly factor ATPAF2 in *Ndufs4* KO hearts, suggesting OXPHOS system structural adaptations in response to cardiac CI dysfunction.

On a metabolic level, *Ndufs4* KO hearts displayed profound alterations. Under normal physiological conditions, the metabolically flexible heart is chiefly reliant on mitochondrial OXPHOS for energy production, with fatty acids and lactic acid as primary fuel sources. During periods of heightened energy demand, stress, or metabolic adaptation, glucose, ketone bodies and to a lesser extent, amino acids can be utilised as alternative fuel sources [62, 63, 64]. In *Ndufs4* KO hearts, there were marked reductions in both primary (long‐chain fatty acids and lactic acid) and alternative energy‐generating substrates (glucose and 3‐hydroxybutyric acid), including key anaplerotic amino acids (glutamine, glutamic acid and alanine). This was accompanied by reductions in some TCA cycle intermediates, most notably succinic acid and other metabolites (DMG, sarcosine, glycerol‐3‐phosphate) capable of fuelling the respiratory chain ubiquinone pool independently of CI. Additionally, elevated levels of 3‐methylhistidine, produced from myocyte protein breakdown, suggest increased reliance on endogenous protein stores. Together, these adaptations allude to a metabolic environment under significant energy stress, with substantial rewiring of both primary and alternative cardiac energy‐generating pathways.

Comparatively, skeletal muscles [46] from this cohort mirrored several metabolic alterations in the heart, although with consistently lower magnitude, suggesting a shared bioenergetic response across contractile tissues. These changes included reductions in lactate, select lipid metabolites (C16:0, glycerol, short to medium‐chain acylcarnitines) and the same anaplerotic amino acids, TCA cycle intermediates and FADH_2_‐linked substrates [46]. In contrast, although some FADH_2_‐linked substrates (succinate, 2‐hydroxyglutarate, glycerol‐3‐phosphate) were similarly decreased in both brain regions and liver, broader metabolic profiles diverged sharply from muscle [46, 48]. Brain regions, particularly the olfactory bulbs, exhibited clear evidence of NAD^+^/NADH imbalance and OXPHOS dysfunction, characterised by elevated levels of glucose, glycolytic and pentose phosphate pathway intermediates, pyruvate, lactate, alanine, branched‐chain amino acids (BCAAs) and fatty acids (C16:0, C18:1, C20:4 *ω*‐6). Conversely, the liver appeared to preserve redox balance and bioenergetic homeostasis, displaying unaltered lactate and lipid metabolite levels but elevated levels of most amino acids, select TCA cycle intermediates (fumarate and malate), UDP‐derivatives of glucose and several purine/pyrimidine metabolites. Together, these metabolic profiles indicate similar bioenergetic stress responses in skeletal and cardiac muscle, significant reductive stress and bioenergetic deficiency in specific brain regions and relative metabolic resilience in the liver.

Abrosimov and colleagues [65] recently demonstrated that CI deficiency triggers a systemic rewiring of fatty acid metabolism, which is ATP‐costly to adipocytes but maintains redox balance and energy homeostasis in hepatocytes. This inter‐organ shuttle bypasses the NADH‐generating steps of glycolysis and the TCA cycle, instead channelling carbon flux through the pentose phosphate pathway to generate NADPH for fatty acid synthesis and export from adipose tissue. In parallel, hepatic fatty acid β‐oxidation increases, producing FADH_2_ to sustain the ubiquinone pool via the electron‐transferring flavoprotein (ETF) complex. Excess hepatic NADH is then exported as lactate and 3‐hydroxybutyrate, which are subsequently utilised by adipose tissue for lipogenesis [65]. Consistent with this model, proteomics of *Ndufs4* KO mice revealed upregulation of enzymes involved in mitochondrial and peroxisomal fatty acid oxidation, as well as ketogenesis, in the liver [66]. A conceptually similar, though intracellular, β‐oxidation shuttle has also been reported in cancer cells. In that context, perturbations in mitochondrial redox balance or partial inhibition of the TCA cycle redirect β‐oxidation intermediates into the citrate–malate shuttle, allowing concurrent fatty acid synthesis and oxidation [67].

In the *Ndufs4* KO heart, it remains unclear whether comparable inter‐organ or intracellular fatty acid oxidation shuttles operate. Nonetheless, the pronounced depletion of cardiac fatty acids, acylcarnitines, glycerol and malate suggests significant rewiring of lipid metabolism. This is supported by proteomic evidence in *Ndufs4* KO hearts [66] indicating a coordinated upregulation of enzymes involved in both fatty acid synthesis and peroxisomal oxidation. Whether this metabolic remodelling serves an adaptive role in maintaining cardiac energetics or instead contributes to progressive cardiac dysfunction remains to be determined. Given the established physical and functional associations between fatty acid β‐oxidation enzymes and respiratory chain supercomplexes [68], targeted investigation of these interactions could clarify whether CI instability constrains mitochondrial β‐oxidation flux in specific tissues, thereby promoting a compensatory shift toward peroxisomal oxidation, which—unlike mitochondrial β‐oxidation—produces hydrogen peroxide.

As previously observed in the skeletal muscle of this cohort [47], the most notable alteration in *Ndufs4* KO hearts was a decrease in DMG, a methylation cycle intermediate and key glycine precursor. Depletion of DMG, along with other glycine precursors such as sarcosine, threonine and 4‐hydroxyproline, suggests increased turnover to meet glycine demands. Glycine is essential for antioxidant defence through the synthesis of the glutathione tripeptide composed of glutamic acid, cysteine and glycine [69, 70]. Marked reductions in glutamic acid and its immediate precursor in the γ‐glutamyl cycle, pyroglutamic acid, could reflect increased glutathione demand in response to oxidative stress, consistent with previous findings of oxidative stress and decreased glutathione in the hearts of whole‐body *Ndufs4* KO mice [6, 7]. While decreases in several glycine precursors (DMG, sarcosine and threonine) were specific to heart, skeletal muscle and urine in this cohort, 4‐hydroxyproline was consistently reduced across all sample types [46, 48]. This systemic reduction warrants further investigation of collagen turnover, prolyl‐4‐hydroxylase activity and hydroxyproline catabolism to elucidate underlying mechanisms. Glutamic acid and pyroglutamic acid showed a similar pattern, decreasing in all tissues except liver where glutathione levels were elevated [46, 48]—likely reflecting the liver's superior capacity to maintain homeostasis during CI deficiency.

Moreover, many alterations in amino acid metabolism in *Ndufs4* KO hearts were centred around glutamic acid pathways. Notably, significant increases in γ‐aminobutyric acid (GABA) levels could signify a shift in glutamic acid usage towards the GABA shunt, which allows energy production through CII while bypassing NAD^+^‐dependent steps of the TCA cycle. This seems to be a heart‐specific response as GABA elevation was not mirrored in other tissues of this cohort, with GABA levels only markedly decreased in urine [46, 48]. Glutamic acid also serves as a precursor to *N*‐acetyl glutamic acid (NAG), a critical activator of the urea cycle that influences nitrogen handling and nitric oxide production. Reductions in the levels of both NAG and citrulline further indicate altered glutamic acid handling in *Ndufs4* KO hearts, which could influence cardiac health. Disruptions in glutamic acid metabolism may affect heart rhythm stability in *Ndufs4* KO mice as glutamic acid contributes to the regulation of myocardial contractility by driving local calcium release in sinoatrial node pacemaker cells (SANPCs) [71]. Interestingly, SANPCs exhibit properties akin to glutamatergic neurons [72], the neuronal subtype shown to be a central driver of CI deficiency‐related encephalopathy in *Ndufs4* KO mice [4]. Considering the role of glutamic acid metabolism in energy production and calcium handling, the potential link between disrupted glutamic acid homeostasis, metabolic dysfunction and arrhythmias in *Ndufs4* KO hearts warrants further investigation through targeted studies. Widespread alterations in glutamate metabolism were evident in this cohort, with skeletal muscle and urine mirroring the NAG and citrulline reductions seen in the heart [46, 48]. Contrastingly, these metabolites remained unchanged in the liver, while most brain regions showed elevated NAG with concomitantly reduced citrulline—highlighting tissue‐specific adaptations in nitrogen and amino acid metabolism [46, 48].

Consistent with the changes in key energy‐generating substrates, we observed significantly elevated IMP and decreased AMP levels in *Ndufs4* KO hearts. This suggests an adaptive shift toward AMP deamination, which enables a rapid breakdown of ATP precursors, maintaining IMP in a readily salvageable pool that can be converted back to AMP and, ultimately, ATP. This mechanism alleviates immediate energy stress and supports contractile function by preventing ADP accumulation and preserving phosphorylation potential but does not effectively support long‐term contractility as it ultimately requires energy investment via the purine salvage pathway [73, 74]. Although these alterations were not evident in skeletal muscles, liver exhibited significant accumulation of IMP and several other purines/pyrimidines, while brain regions showed some variable alterations in purine metabolism [46, 48].

The most significant increase in metabolite levels was observed in the non‐proteinogenic amino acid, pipecolic acid. This lysine catabolite is produced predominantly in the liver and has emerged as a key immunomodulator that protects against oxidative stress [75] and exerts anti‐inflammatory properties [76] through its regulation of mechanistic target of rapamycin complex (mTORC). In addition to the heart, we observed a consistent and significant accumulation of pipecolic acid and depletion of downstream metabolite 2‐aminoadipic acid in skeletal muscles [47], liver [48] and brain regions [45] of this *Ndufs4* KO cohort. These findings suggest that systemic alterations in lysine catabolism may play a critical role in the metabolic and inflammatory perturbations associated with CI dysfunction.

Another non‐proteinogenic amino acid, BAIBA, was significantly elevated in *Ndufs4* KO hearts. BAIBA is primarily produced from the catabolism of the BCAA valine in skeletal muscle, with its accumulation seen in skeletal and cardiac muscle during periods of heightened energy demand (i.e., exercise). In this context, BAIBA acts as a myokine, promoting fatty acid uptake and oxidation while exerting anti‐inflammatory, antioxidant, as well as cardioprotective effects [77, 78]. In *Ndufs4* KO mice, the accumulation of BAIBA could reflect adaptive responses to significant metabolic stress. This seems to be specific to the heart, as BAIBA could not be reliably detected in other tissues from this cohort, while its urinary excretion was significantly decreased [46].

Taken together, several key candidate pathways have been identified for further mechanistic investigation in LS; however, steady‐state metabolomics alone cannot definitively distinguish pathophysiological drivers from secondary metabolic consequences. Future studies employing stable isotope tracing and targeted metabolite challenges in *Ndufs4* KO mice or derived cells will be critical to differentiate adaptive metabolic remodelling from secondary consequences of mitochondrial dysfunction. Additionally, as this study employed a single‐sex design, the generalisability of the findings to female mice may be limited. Thus, inclusion of both sexes in future work will be important to assess potential sex‐specific metabolic differences in these pathways.

## 5 Conclusions

This study provides a comprehensive analysis of the metabolic disruptions caused by CI deficiency in the heart of severely diseased whole‐body *Ndufs4* KO mice. We observed near‐complete CI loss, accompanied by significant reductions in downstream respiratory complex activities, leading to significant CI functional loss and a compensatory shift towards CII‐driven respiration. Metabolic profiling confirms a profound disturbance in mitochondrial bioenergetics, with severely reduced levels of primary and alternative cardiac energy‐generating fuels. Notably, altered amino acid metabolism, particularly involving DMG and glutamic acid related metabolites, indicates a shift in cellular energy‐generating pathways, nitrogen handling and antioxidant defence mechanisms. Additionally, the accumulation of metabolites such as pipecolic acid and BAIBA suggests systemic and cardiac‐specific metabolic adaptations, which point to an intricate interplay between energy production, oxidative stress and inflammation that may contribute to the pathophysiology of CI deficiency in cardiac tissue. Future studies targeting these metabolic pathways will be crucial in understanding their precise role in disease progression and their potential as therapeutic targets to mitigate cardiac dysfunction in CI‐related mitochondrial diseases.

## Author Contributions

**Karin Terburgh:** conceptualisation, visualisation, writing – original draft preparation, co‐supervision. **Nastassja Sweeney:** conceptualisation, methodology, investigation, formal analysis, writing – reviewing and editing. **Roan Louw:** conceptualisation, investigation, resources, writing – reviewing and editing, supervision, funding acquisition.

## Funding

This work was supported by the National Research Foundation of South Africa (NRF Grant no 120829) and the North‐West University (NWU). Opinions expressed and conclusions arrived at are those of the authors and are not necessarily to be attributed to the NRF or NWU.

## Conflicts of Interest

The authors declare no conflicts of interest.

## Supporting information


**Table S1:** Reports of hypertrophic cardiomyopathy (HCM) in patients with pathogenic *NDUFS4* mutations.
**Table S2:** dMRM conditions for compounds measured via LC‐MS/MS.

## Data Availability

The data from this study [79] are available through the NIH Common Fund's Data Repository and Coordinating Center, supported by NIH grant U01‐DK097430, at the Metabolomics Workbench website (http://www.metabolomicsworkbench.org) under the study ID, ST003935.
