# Supplementary material for: Energy Metabolism Under Stress: Late‐Stage Leigh Syndrome Reveals Profound Cardiometabolic Perturbations in Ndufs4 KO Mice
Source: J Inherit Metab Dis. 2026 Jan 14;49(1):e70142. doi: 10.1002/jimd.70142 (PMC12801098; doi:10.1002/jimd.70142)
Supplement: Supplementary file 1 — Table S1: Reports of hypertrophic cardiomyopathy (HCM) in patients with pathogenic NDUFS4 mutations. Table S2: dMRM conditions for compounds measured via LC‐MS/MS. [file JIMD-49-0-s001.docx]

**Supplementary information**

________________________________________________________________________________

**Energy metabolism under stress: late-stage Leigh syndrome reveals profound cardiometabolic perturbations in *Ndufs4* KO mice**

Karin Terburgh, Nastassja Sweeney & Roan Louw

________________________________________________________________________________

**Table S1: Reports of hypertrophic cardiomyopathy (HCM) in patients with pathogenic *NDUFS4* mutations**

| Case report reference | *NDUFS4* patients | HCM cases |
| --- | --- | --- |
| van den Heuvel, Ruitenbeek [1] | 1 | 0 |
| Budde, van den Heuvel [2] | 2 | 1 |
| Petruzzella, Vergari [3] | 1 | 1 |
| Budde, van den Heuvel [4] | 1 | 0 |
| Bénit, Steffann [5] | 2 | 0 |
| Anderson, Chung [6] | 3 | 1 |
| Leshinsky-Silver, Lebre [7] | 1 | 0 |
| Calvo, Tucker [8] | 2 | 0 |
| Assouline, Jambou [9] * | 5 | 2 |
| Haack, Madignier [10] | 1 | 1 |
| Lombardo, Ceglia [11] | 1 | 0 |
| Assereto, Robbiano [12] | 2 | 0 |
| Ortigoza-Escobar, Oyarzabal [13] * | 1 | 0 |
| Lamont, Beaulieu [14] | 2 | 2 |
| Bris, Rouaud [15] | 1 | 0 |
| Sage-Schwaede, Engelstad [16] | 1 | 0 |
| González-Quintana, Trujillo-Tiebas [17] | 1 | 0 |
| Vafaee-Shahi, Ghasemi [18] | 1 | 0 |
| Total | **32** | **8** |

*In their reviews, Assouline *et al.* (2012) and Ortigoza-Escobar *et al.* (2016) both noted at total of 5 HCM cases reported out of 18 patients (from 15 families) and 22 patients (from 18 families), respectively.

**Metabolomics methodology**

**Internal standard preparation**

An internal standard (IS) mixture comprising N,N-dimethyl-phenylalanine (DMPA), 3-phenylbutyric acid (3-PBA), and norleucine (each 309.89 ppm from 10 000 ppm stock solutions) was added to each sample at 1 µg per mg tissue to correct for extraction bias. Norleucine was used for normalisation in GC-TOF-MS, and DMPA served as the IS for both LC-MS/MS and ¹H-NMR analyses.

For LC-MS/MS, an additional stable-isotope IS mixture (2.5 ppm in water, 200 µL per sample) was added prior to derivatisation. This mixture contained L-valine-d8, L-isoleucine-d10, L-phenylalanine-d5, L-lysine-d4, L-glutamine-^13^C_5_^15^N_2_, L-asparagine_^13^C_4_N^15^N_2_:H₂O, L-arginine_d4, L-citrulline_d4, and L-carnitine-methyl-d3·HCl, octanoyl-L-carnitine-8,8,8d3·HCl, dodecanoyl-L-carnitine-10,10,10 d3·HCl, and octadecanoylLcarnitine-18,18,18d3·HCl to account for derivatisation and ionisation variability.

**Metabolite extraction from heart tissue**

Metabolites were extracted from mouse heart tissue using a modified single-phase Bligh–Dyer method [19] with a methanol:water:chloroform ratio of 3:1:1. On a chilled, sanitised surface, tissues were rinsed in PBS, blotted dry, and weighed (40–75 mg per sample). Each sample was placed in a safe-lock tube, followed by 12 µL methanol per mg tissue, the IS mixture (1 µg/mg), and 3.227 µL H₂O per mg tissue. Samples were vortexed and homogenised using three 3 mm tungsten carbide/stainless-steel beads for 2 min at 30 Hz. After bead removal, 1 mL chloroform was added, and the samples were vortexed (1 min), incubated on ice (10 min), and centrifuged (20 000 × g, 10 min, 4 °C). The resulting supernatant was transferred to new tubes for platform-specific analysis.

**Quality control samples**

Heart extracts were pooled to generate QC samples analysed alongside each batch. For GC-TOF-MS and LC-MS/MS, five QC samples were included per batch (at the beginning, end, and evenly distributed throughout), while for ¹H-NMR, three QC samples were analysed per batch (at the beginning, middle, and end).

**GC-TOF-MS derivatisation and analysis**

Heart extracts (500 µL) were dried under nitrogen (37 °C, 15 min) and derivatised by sequential oximation and silylation. Oximation was performed with 50 µL of methoxyamine hydrochloride (20 mg/mL in pyridine) for 60 min at 60 °C. After cooling, 50 µL of BSTFA + 1 % TMCS was added for silylation (60 min, 60 °C). Following cooling, 100 µL of the derivatised extract was transferred to vials for analysis.

For untargeted GC-TOF-MS, chromatographic separation was achieved using a Rxi®-5Sil MS capillary column (28.6 m × 250 µm × 0.25 µm; Restek, Cat. no. 13620) on an Agilent 7890A gas chromatograph equipped with an Agilent 7693 autosampler and coupled to a LECO Pegasus HT time-of-flight mass analyser fitted with an electron-impact (EI) ionisation source. ChromaTOF software (v 4.5x; LECO Corporation) was used for data acquisition and processing.

Samples (1 µL) were injected at a split ratio of 1:5. The GC oven was held at 70 °C (1 min), then ramped 7 °C/min to 120 °C, 10 °C/min to 230 °C, and 13 °C/min to 300 °C (hold 1 min), giving a ~25.5 min run. The transfer line and ion source were maintained at 225 °C and 200 °C, respectively. EI ionisation was conducted at −70 V with a 250 s solvent delay. Spectra were acquired at 20 Hz across m/z 50–950. Peaks were annotated via spectral and retention time matching to commercial (NIST11, mainlib and replib) and in-house libraries, with a similarity setting of at least 80%.

**LC-MS/MS derivatisation and analysis**

Cardiac tissue extracts (100 µL) were evaporated under a gentle nitrogen stream at 37 °C for 15 min prior to derivatisation by butylation. Fresh butanolic-HCl reagent was prepared by adding acetyl chloride to 1-butanol (4:1, v/v) on ice. Each dried extract was treated with 300 µL of this reagent and incubated at 50 °C for 60 min. The reaction mixtures were subsequently dried again (37 °C, 60 min) and reconstituted in 100 µL of water–acetonitrile (1:1, v/v) containing 1 % formic acid. Samples were vortexed, centrifuged at 20 000 × g for 20 min at 4 °C, and 70 µL of the clarified supernatant was transferred to tapered glass inserts (250 µL) in glass vials for LC-MS/MS analysis.

Semi-targeted profiling of amino acids and acylcarnitines was carried out following the method of du Toit et al. [20], with modifications to enable operation in dynamic multiple reaction monitoring (dMRM) mode. Analyses were performed using an Agilent 1260 Infinity LC system coupled to a 6470 Triple Quadrupole MS equipped with a Jet Stream electrospray ionisation source (Agilent Technologies, USA). Optimal dMRM parameters were established using Agilent MassHunter MS Optimizer (v B02.01), and data acquisition and processing were conducted in Agilent MassHunter Workstation (v B10.00).

Chromatographic separation was achieved on a Zorbax SB-Aq 80 Å StableBond column (100 mm × 2.1 mm, 1.8 µm; Agilent Technologies) fitted with a Zorbax Eclipse Plus C18 guard column (5 mm × 2.1 mm, 1.8 µm). The column temperature was maintained at 45 °C. Injections of 0.5 µL were eluted using a binary gradient of solvent A (water + 0.1 % formic acid) and solvent B (acetonitrile + 0.1 % formic acid) as follows: 0–0.2 min, 95 % A; 0.2–2.0 min, linear increase to 25 % B; 2.0–7.0 min, hold 25 % B; 7.0–7.5 min, ramp to 90 % B; 7.5–9.1 min, hold 90 % B; 9.1–12.0 min, increase to 95 % B; 12.0–13.0 min, return to 5 % B; and re-equilibration to complete a 16-min total runtime. The mobile-phase flow rate was maintained at 0.3 mL/min for the first 9 min, increased to 0.4 mL/min over 0.1 min, and held for 3.9 min.

The mass spectrometer operated in positive ESI mode with dMRM acquisition using optimised transitions (Table S2) based on authentic reference compounds. Source parameters were as follows: nitrogen drying gas at 280 °C and 9 L/min, nebuliser pressure at 45 psi, sheath gas at 400 °C and 12 L/min, capillary voltage 4500 V, and nozzle voltage 500 V. To ensure confident metabolite identification, two diagnostic transitions were monitored per analyte, permitting confirmation by both fragmentation pattern and retention time [21, 22]. A reference standard mixture was analysed alongside study samples for further validation of compound identities.

**Table S2: dMRM conditions for compounds measured via LC-MS/MS**

| **Analyte name** | **Quantifier / qualifier** | **Precursor ion (m/z)** | **Product ion (m/z)** | **F (V)** | **CE (V)** | **Retention time (min)** |
| --- | --- | --- | --- | --- | --- | --- |
| 1-Methylhistidine | Quantifier | 226.4 | 124.1 | 93 | 20 | 1.39 |
| 1-Methylhistidine | Qualifier | 226.4 | 83.1 | 93 | 36 | 1.39 |
| 2-Aminoadipic acid | Qualifier | 274.2 | 172.1 | 106 | 16 | 8.28 |
| 2-Aminoadipic acid | Quantifier | 274.2 | 98.1 | 106 | 24 | 8.28 |
| β-Alanine | Qualifier | 146.2 | 90.1 | 68 | 8 | 2.47 |
| β-Alanine | Quantifier | 146.2 | 72.1 | 68 | 8 | 2.47 |
| 3-Aminoisobutyric acid | Quantifier | 160.1 | 86.1 | 73 | 8 | 3.39 |
| 3-Aminoisobutyric acid | Qualifier | 160.1 | 57.2 | 73 | 20 | 3.39 |
| 3-Hydroxykynurenine | Quantifier | 281.2 | 152.0 | 88 | 16 | 4.67 |
| 3-Hydroxykynurenine | Qualifier | 281.2 | 110.0 | 88 | 28 | 4.67 |
| 3-Methylhistidine | Quantifier | 226.4 | 96.1 | 111 | 32 | 1.29 |
| 3-Methylhistidine | Qualifier | 226.4 | 95.1 | 111 | 52 | 1.29 |
| 4-Aminobutyric acid | Quantifier | 160.1 | 87.1 | 73 | 12 | 3.19 |
| 4-Aminobutyric acid | Qualifier | 160.1 | 86.3 | 73 | 12 | 3.19 |
| 4-Hydroxyproline | Quantifier | 188.1 | 86.1 | 98 | 20 | 2.50 |
| 4-Hydroxyproline | Qualifier | 188.1 | 68.1 | 98 | 36 | 2.50 |
| Acetylcarnitine (C2) | Qualifier | 260.2 | 145.0 | 101 | 16 | 4.37 |
| Acetylcarnitine (C2) | Quantifier | 260.2 | 85.1 | 101 | 24 | 4.37 |
| Acetylcarnitine (C2)_IS | Qualifier | 263.2 | 148.0 | 106 | 16 | 4.36 |
| Acetylcarnitine (C2)_IS | Quantifier | 263.2 | 85.1 | 106 | 24 | 4.36 |
| Alanine | Quantifier | 146.1 | 90.1 | 70 | 8 | 2.47 |
| Alanine | Qualifier | 146.1 | 57.2 | 70 | 12 | 2.47 |
| Arginine | Quantifier | 231.2 | 70.1 | 111 | 36 | 1.28 |
| Arginine | Qualifier | 231.2 | 60.2 | 111 | 16 | 1.28 |
| Asparagine | Qualifier | 189.1 | 87.1 | 73 | 12 | 1.60 |
| Asparagine | Quantifier | 189.1 | 74.1 | 73 | 20 | 1.60 |
| Aspartic acid | Quantifier | 246.2 | 144.0 | 88 | 12 | 6.33 |
| Aspartic acid | Qualifier | 246.2 | 88.1 | 88 | 20 | 6.33 |
| Butyrylcarnitine (C4) | Qualifier | 288.2 | 173.0 | 111 | 16 | 6.31 |
| Butyrylcarnitine (C4) | Quantifier | 288.2 | 85.0 | 111 | 24 | 6.31 |
| Carnitine (C0) | Quantifier | 218.2 | 103.0 | 106 | 20 | 3.35 |
| Carnitine (C0) | Qualifier | 218.2 | 85.0 | 106 | 28 | 3.35 |
| Citrulline | Quantifier | 232.2 | 215.0 | 83 | 12 | 2.73 |
| Citrulline | Qualifier | 232.2 | 70.1 | 83 | 36 | 2.73 |
| Creatine | Qualifier | 188.1 | 132.0 | 88 | 16 | 3.41 |
| Creatine | Quantifier | 188.1 | 90.1 | 88 | 20 | 3.41 |
| Cystathionine | Quantifier | 335.2 | 190.0 | 126 | 16 | 3.93 |
| Cystathionine | Qualifier | 335.2 | 88.1 | 126 | 36 | 3.93 |
| Cystine | Quantifier | 353.2 | 130.1 | 98 | 20 | 3.93 |
| Cystine | Qualifier | 353.2 | 74.1 | 98 | 40 | 3.93 |
| Decanoylcarnitine (C10) | Qualifier | 372.3 | 257.1 | 144 | 20 | 9.60 |
| Decanoylcarnitine (C10) | Quantifier | 372.3 | 85.1 | 144 | 32 | 9.60 |
| Dimethylglycine | Qualifier | 160.3 | 104.1 | 78 | 12 | 2.36 |
| Dimethylglycine | Quantifier | 160.3 | 58.2 | 78 | 24 | 2.36 |
| Dimethylphenylalanine | Quantifier | 250.1 | 148.1 | 106 | 24 | 6.77 |
| Dimethylphenylalanine | Qualifier | 250.1 | 133.1 | 106 | 40 | 6.77 |
| Dodecanoylcarnitine (C12) | Qualifier | 400.3 | 285.2 | 159 | 20 | 9.70 |
| Dodecanoylcarnitine (C12) | Quantifier | 400.3 | 85.1 | 159 | 32 | 9.70 |
| Glutamic acid | Qualifier | 260.2 | 158.1 | 98 | 16 | 7.05 |
| Glutamic acid | Quantifier | 260.2 | 84.1 | 98 | 28 | 7.05 |
| Glutamine | Qualifier | 203.1 | 130.0 | 83 | 16 | 2.02 |
| Glutamine | Quantifier | 203.1 | 84.1 | 83 | 24 | 2.02 |
| Glycine | Quantifier | 132.1 | 76.1 | 65 | 4 | 1.85 |
| Glycine | Qualifier | 132.1 | 57.2 | 65 | 12 | 1.85 |
| Hexadecanoylcarnitine (C16) | Qualifier | 456.4 | 341.2 | 172 | 24 | 10.0 |
| Hexadecanoylcarnitine (C16) | Quantifier | 456.4 | 85.0 | 172 | 32 | 10.0 |
| Hexanoylcarnitine (C6) | Qualifier | 316.2 | 201.0 | 121 | 16 | 9.3 |
| Hexanoylcarnitine (C6) | Quantifier | 316.2 | 85.1 | 121 | 24 | 9.3 |
| Histidine | Quantifier | 212.1 | 110.0 | 98 | 20 | 1.09 |
| Histidine | Qualifier | 212.1 | 83.1 | 98 | 40 | 1.09 |
| Isoleucine | Qualifier | 188.2 | 86.1 | 83 | 12 | 4.81 |
| Isoleucine | Quantifier | 188.2 | 69.2 | 83 | 28 | 4.81 |
| Isoleucine_IS | Quantifier | 198.2 | 96.2 | 78 | 16 | 4.81 |
| Isoleucine_IS | Qualifier | 198.2 | 78.2 | 78 | 28 | 4.81 |
| Isovalerylcarnitine (C5) | Qualifier | 302.2 | 187.0 | 121 | 16 | 8.37 |
| Isovalerylcarnitine (C5) | Quantifier | 302.2 | 85.1 | 121 | 28 | 8.37 |
| Kynurenine | Quantifier | 265.4 | 136.0 | 98 | 16 | 5.29 |
| Kynurenine | Qualifier | 265.4 | 118.0 | 98 | 36 | 5.29 |
| Leucine | Quantifier | 188.2 | 86.1 | 78 | 12 | 4.81 |
| Leucine | Qualifier | 188.2 | 57.2 | 78 | 24 | 4.81 |
| Lysine | Quantifier | 203.1 | 84.1 | 88 | 24 | 1.3 |
| Lysine | Qualifier | 203.1 | 56.2 | 88 | 56 | 1.3 |
| Lysine_IS | Quantifier | 207.2 | 88.2 | 93 | 24 | 1.3 |
| Lysine_IS | Qualifier | 207.2 | 56.2 | 93 | 52 | 1.3 |
| Methionine | Quantifier | 206.1 | 104.0 | 78 | 12 | 4.39 |
| Methionine | Qualifier | 206.1 | 56.2 | 78 | 20 | 4.39 |
| N-Acetylaspartic acid (NAA) | Quantifier | 288.2 | 144.1 | 88 | 20 | 9.3 |
| N-Acetylaspartic acid (NAA) | Qualifier | 288.2 | 88.1 | 88 | 32 | 9.3 |
| N-Acetylglutamic acid (NAG) | Quantifier | 302.2 | 186.0 | 93 | 16 | 9.3 |
| N-Acetylglutamic acid (NAG) | Qualifier | 302.2 | 84.1 | 93 | 32 | 9.3 |
| Octadecanoylcarnitine (C18) | Quantifier | 484.0 | 85.1 | 162 | 36 | 11.00 |
| Octadecanoylcarnitine (C18) | Qualifier | 484.0 | 57.2 | 162 | 76 | 11.00 |
| Octadecanoylcarnitine (C18)_IS | Quantifier | 487.5 | 85.1 | 162 | 36 | 11.00 |
| Octadecanoylcarnitine (C18)_IS | Qualifier | 487.5 | 57.2 | 162 | 80 | 11.00 |
| Octanoylcarnitine (C8) | Qualifier | 344.3 | 229.1 | 134 | 20 | 9.50 |
| Octanoylcarnitine (C8) | Quantifier | 344.3 | 85.1 | 134 | 28 | 9.50 |
| Octanoylcarnitine (C8)_IS | Qualifier | 347.3 | 232.1 | 129 | 20 | 9.50 |
| Octanoylcarnitine (C8)_IS | Quantifier | 347.3 | 85.1 | 129 | 28 | 9.50 |
| Ornithine | Qualifier | 189.2 | 172.1 | 83 | 8 | 1.20 |
| Ornithine | Quantifier | 189.2 | 70.1 | 83 | 24 | 1.20 |
| Phenylalanine | Quantifier | 222.2 | 120.1 | 93 | 16 | 5.62 |
| Phenylalanine | Qualifier | 222.2 | 103.0 | 93 | 44 | 5.62 |
| Phenylalanine_IS | Quantifier | 227.2 | 125.1 | 88 | 16 | 5.41 |
| Phenylalanine_IS | Qualifier | 227.2 | 106.1 | 88 | 44 | 5.41 |
| Pipecolic acid | Quantifier | 186.1 | 84.1 | 103 | 20 | 3.78 |
| Pipecolic acid | Qualifier | 186.1 | 56.2 | 103 | 52 | 3.78 |
| Proline | Qualifier | 172.1 | 116.0 | 88 | 12 | 3.28 |
| Proline | Quantifier | 172.1 | 70.1 | 88 | 20 | 3.28 |
| Propionylcarnitine (C3) | Qualifier | 274.2 | 159.0 | 111 | 16 | 4.98 |
| Propionylcarnitine (C3) | Quantifier | 274.2 | 85.1 | 111 | 24 | 4.98 |
| Pyroglutamic acid | Quantifier | 186.1 | 84.1 | 68 | 20 | 5.12 |
| Pyroglutamic acid | Qualifier | 186.1 | 130.0 | 68 | 12 | 5.12 |
| Sarcosine | Quantifier | 146.1 | 90.1 | 73 | 8 | 2.06 |
| Sarcosine | Qualifier | 146.1 | 57.2 | 73 | 16 | 2.06 |
| Serine | Qualifier | 162.1 | 106.0 | 78 | 8 | 1.74 |
| Serine | Quantifier | 162.1 | 60.2 | 78 | 12 | 1.74 |
| Tetradecanoylcarnitine (C14) | Qualifier | 428.4 | 313.2 | 149 | 20 | 9.70 |
| Tetradecanoylcarnitine (C14) | Quantifier | 428.4 | 85.1 | 149 | 32 | 9.70 |
| Threonine | Quantifier | 176.1 | 74.1 | 73 | 16 | 2.35 |
| Threonine | Qualifier | 176.1 | 56.2 | 73 | 28 | 2.35 |
| Trimethylglycine | Quantifier | 174.2 | 118.0 | 103 | 20 | 3.18 |
| Trimethylglycine | Qualifier | 174.2 | 58.2 | 103 | 52 | 3.18 |
| Tryptophan | Quantifier | 261.2 | 244.1 | 88 | 8 | 6.32 |
| Tryptophan | Qualifier | 261.2 | 132.0 | 88 | 32 | 6.32 |
| Tyrosine | Quantifier | 238.2 | 136.0 | 88 | 16 | 4.37 |
| Tyrosine | Qualifier | 238.2 | 91.1 | 88 | 48 | 4.37 |
| Valine | Quantifier | 174.2 | 72.2 | 73 | 12 | 4.11 |
| Valine | Qualifier | 174.2 | 55.2 | 73 | 36 | 4.11 |
| Valine_IS | Quantifier | 182.2 | 80.2 | 73 | 16 | 4.10 |
| Valine_IS | Qualifier | 182.2 | 62.2 | 73 | 40 | 4.10 |

*Conditions for butylated compounds and deuterium-labelled internal standards (IS) are detailed together with their precursor- and product ions. The collision cell accelerator voltage for all compounds was 4 V. Abbreviations: CE, collision energy voltage; F, fragmentor voltage; m/z, mass to charge ratio; V, Voltage.*

**¹H-NMR sample preparation and analysis**

Untargeted metabolite profiling of cardiac extracts was performed using the miniaturised ¹H-NMR method described by Mason et al. [23], optimised for small-volume biological samples. Spectra were acquired on a 500 MHz Bruker Avance™ III HD NMR spectrometer equipped with a 5 mm triple-resonance inverse (TXI) [¹H, ¹⁵N, ¹³C] probe optimised for proton detection and a SampleXpress autosampler (Bruker, Germany). Data acquisition and primary processing were conducted using Bruker TopSpin software (v 3.5), while subsequent spectral handling and metabolite assignment were performed in Bruker AMIX (v 3.9.14).

Heart extracts (250 µL) were evaporated under nitrogen at 37 °C, reconstituted in 60 µL of HPLC-grade water, and centrifuged at 12 000 × g for 5 min at 25 °C to remove macromolecular components that could interfere with spectral clarity. Using a programmed electronic pipetting sequence, 6 µL of TSP-d₄ NMR buffer and 54 µL of supernatant (final D₂O:H₂O ratio of 10:90 %) were aspirated and dispensed into 2 mm MATCH™ NMR tubes (outer Ø 2.0 mm, inner Ø 1.6 mm, 100 mm length). The mixture was homogenised by a single aspirate–dispense cycle. Between samples, the pipetting needle was rinsed with three 100 µL volumes of Milli-Q water to prevent cross-contamination. Prepared tubes were then assembled with MATCH™ gripper adaptors and placed in the autosampler for analysis.

Automated pre-acquisition adjustments were applied to ensure spectral reproducibility, including shimming to the TSP resonance for magnetic field homogeneity, locking to the D₂O signal for drift correction, probe tuning and matching, and pulse calibration. Water suppression was achieved using a NOESY-presat pulse sequence with an 8 µs 90° excitation pulse and a 4 s relaxation delay. Samples were maintained at 300 K, and spectra were collected with 128 transients over 32 K data points at a spectral width of 6 000 Hz. Each scan employed an 8 µs 90° pulse, a 4 s relaxation delay, a receiver gain of 64, and a 2.72 s acquisition time. Fourier transformation, baseline correction, and phase adjustment were executed automatically, and the TSP resonance was referenced to 0.00 ppm. Spectral quality was verified manually by ensuring TSP and metabolite line widths remained <1 Hz.

Metabolite identification was based on comparison of 1D ¹H-NMR spectra to reference spectra from both commercial and in-house compound libraries. Structural confirmation was achieved through analysis of 2D correlation spectroscopy (COSY) and J-resolved (JRES) ¹H–¹H NMR data, ensuring high confidence in metabolite assignments [21, 22]. Two-dimensional spectra were acquired on pooled samples representing all 1D-analyzed extracts, using the same instrument under the following parameters: 16 scans per increment, 8 000 Hz spectral width per dimension, 2 s recycle delay, and an 8.5 µs excitation pulse.

**References**

[1] van den Heuvel L, Ruitenbeek W, Smeets R, Gelman-Kohan Z, Elpeleg O, Loeffen J, et al. Demonstration of a new pathogenic mutation in human complex I deficiency: a 5-bp duplication in the nuclear gene encoding the 18-kD (AQDQ) subunit. Am J Hum Genet. 1998;62:262-8.

[2] Budde SM, van den Heuvel LP, Janssen AJ, Smeets RJ, Buskens CA, DeMeirleir L, et al. Combined enzymatic complex I and III deficiency associated with mutations in the nuclear encoded NDUFS4 gene. Biochem Biophys Res Commun. 2000;275:63-8.

[3] Petruzzella V, Vergari R, Puzziferri I, Boffoli D, Lamantea E, Zeviani M, et al. A nonsense mutation in the NDUFS4 gene encoding the 18 kDa (AQDQ) subunit of complex I abolishes assembly and activity of the complex in a patient with Leigh-like syndrome. Hum Mol Genet. 2001;10:529-36.

[4] Budde SMS, van den Heuvel LPWJ, Smeets RJP, Skladal D, Mayr JA, Boelen C, et al. Clinical heterogeneity in patients with mutations in the NDUFS4 gene of mitochondrial complex I. Journal of Inherited Metabolic Disease. 2003;26:813-5.

[5] Bénit P, Steffann J, Lebon S, Chretien D, Kadhom N, de Lonlay P, et al. Genotyping microsatellite DNA markers at putative disease loci in inbred/multiplex families with respiratory chain complex I deficiency allows rapid identification of a novel nonsense mutation (IVS1nt −1) in the NDUFS4 gene in Leigh syndrome. Hum Genet. 2003;112:563-6.

[6] Anderson SL, Chung WK, Frezzo J, Papp JC, Ekstein J, DiMauro S, et al. A novel mutation in NDUFS4 causes Leigh syndrome in an Ashkenazi Jewish family. J Inherit Metab Dis. 2008;31 Suppl 2:S461-7.

[7] Leshinsky-Silver E, Lebre AS, Minai L, Saada A, Steffann J, Cohen S, et al. NDUFS4 mutations cause Leigh syndrome with predominant brainstem involvement. Mol Genet Metab. 2009;97:185-9.

[8] Calvo SE, Tucker EJ, Compton AG, Kirby DM, Crawford G, Burtt NP, et al. High-throughput, pooled sequencing identifies mutations in NUBPL and FOXRED1 in human complex I deficiency. Nat Genet. 2010;42:851-8.

[9] Assouline Z, Jambou M, Rio M, Bole-Feysot C, de Lonlay P, Barnerias C, et al. A constant and similar assembly defect of mitochondrial respiratory chain complex I allows rapid identification of NDUFS4 mutations in patients with Leigh syndrome. Biochim Biophys Acta. 2012;1822:1062-9.

[10] Haack TB, Madignier F, Herzer M, Lamantea E, Danhauser K, Invernizzi F, et al. Mutation screening of 75 candidate genes in 152 complex I deficiency cases identifies pathogenic variants in 16 genes including NDUFB9. J Med Genet. 2012;49:83-9.

[11] Lombardo B, Ceglia C, Tarsitano M, Pierucci I, Salvatore F, Pastore L. Identification of a deletion in the NDUFS4 gene using array-comparative genomic hybridization in a patient with suspected mitochondrial respiratory disease. Gene. 2014;535:376-9.

[12] Assereto S, Robbiano A, Di Rocco M, Rossi A, Cassandrini D, Panicucci C, et al. Functional characterization of the c.462delA mutation in the NDUFS4 subunit gene of mitochondrial complex I. Clin Genet. 2014;86:99-101.

[13] Ortigoza-Escobar JD, Oyarzabal A, Montero R, Artuch R, Jou C, Jiménez C, et al. Ndufs4 related Leigh syndrome: a case report and review of the literature. Mitochondrion. 2016;28:73-8.

[14] Lamont RE, Beaulieu CL, Bernier FP, Sparkes R, Innes AM, Jackel-Cram C, et al. A novel NDUFS4 frameshift mutation causes Leigh disease in the Hutterite population. Am J Med Genet A. 2017;173:596-600.

[15] Bris C, Rouaud T, Desquiret-Dumas V, Gueguen N, Goudenege D, Barth M, et al. Novel NDUFS4 gene mutation in an atypical late-onset mitochondrial form of multifocal dystonia. Neurol Genet. 2017;3:e205.

[16] Sage-Schwaede A, Engelstad K, Salazar R, Curcio A, Khandji A, Garvin Jr JH, et al. Exploring mTOR inhibition as treatment for mitochondrial disease. Ann Clin Transl Neurol. 2019;6:1877-81.

[17] González-Quintana A, Trujillo-Tiebas MJ, Fernández-Perrone AL, Blázquez A, Lucia A, Morán M, et al. Uniparental isodisomy as a cause of mitochondrial complex I respiratory chain disorder due to a novel splicing NDUFS4 mutation. Mol Genet Metab. 2020;131:341-8.

[18] Vafaee-Shahi M, Ghasemi S, Beiraghi Toosi M, Ashrafi MR, Badv RS, Tavasoli AR, et al. Bilateral horizontal gaze palsy in an 8-year-old girl: a rare case with NDUFS4 gene mutation. Clin Case Rep. 2021;9:e04748.

[19] Gullberg J, Jonsson P, Nordström A, Sjöström M, Moritz T. Design of experiments: an efficient strategy to identify factors influencing extraction and derivatization of Arabidopsis thaliana samples in metabolomic studies with gas chromatography/mass spectrometry. Anal Biochem. 2004;331:283-95.

[20] du Toit WL, Kruger R, Gafane-Matemane LF, Schutte AE, Louw R, Mels CMC. Using urinary metabolomics to identify metabolic pathways linked to cardiac structural alterations in young adults: The African-PREDICT study. Nutrition, Metabolism and Cardiovascular Diseases. 2023;33:1574-82.

[21] Schymanski EL, Jeon J, Gulde R, Fenner K, Ruff M, Singer HP, et al. Identifying small molecules via high resolution mass spectrometry: communicating confidence. Environ Sci Technol. 2014;48:2097-8.

[22] Sumner LW, Amberg A, Barrett D, Beale MH, Beger R, Daykin CA, et al. Proposed minimum reporting standards for chemical analysis Chemical Analysis Working Group (CAWG) Metabolomics Standards Initiative (MSI). Metabolomics : Official journal of the Metabolomic Society. 2007;3:211-21.

[23] Mason SW, Terburgh K, Louw R. Miniaturized ^1^H-NMR method for analyzing limited-quantity samples applied to a mouse model of Leigh disease. Metabolomics. 2018;14:1-12.
